# Exploring the Association between Health-Related Physical Fitness and Quality of Life in Patients with Cancer: A Cross-Sectional Study

**DOI:** 10.3390/healthcare12161643

**Published:** 2024-08-17

**Authors:** Anita Borsati, Diana Giannarelli, Lorenzo Belluomini, Christian Ciurnelli, Alessio Colonna, Irene D’Amico, Arianna Daniele, Nicole Del Bianco, Linda Toniolo, Ilaria Trestini, Daniela Tregnago, Jessica Insolda, Marco Sposito, Massimo Lanza, Michele Milella, Federico Schena, Sara Pilotto, Alice Avancini

**Affiliations:** 1 Department of Medicine, University of Verona, 37129 Verona, Italy; anita.borsati_02@univr.it; 2 A. Gemelli University Hospital Foundation, IRCCS-Epidemiology&Biostatistic, 00168 Rome, Italy; diana.giannarelli@policlinicogemelli.it; 3 Section of Innovation Biomedicine—Oncology Area, Department of Engineering for Innovation Medicine (DIMI), University of Verona and University and Hospital Trust (AOUI) of Verona, 37126 Verona, Italy; lorenzo.belluomini@univr.it (L.B.); daniela.tregnago_01@univr.it (D.T.); jessica.insolda@univr.it (J.I.); marcosposito91@gmail.com (M.S.); michele.milella@univr.it (M.M.); sara.pilotto@univr.it (S.P.); 4 Department of Neuroscience, Biomedicine, and Movement Sciences, University of Verona, 37129 Verona, Italyalessio.colonna@studenti.univr.it (A.C.); irene.damico@studenti.univr.it (I.D.); arianna.daniele@studenti.univr.it (A.D.); nicole.delbianco@studenti.univr.it (N.D.B.); linda.toniolo@studenti.univr.it (L.T.); massimo.lanza@univr.it (M.L.); federico.schena@univr.it (F.S.); 5 Dietetics Service, Medical Direction, University Hospital of Verona, 37126 Verona, Italy; ilaria.trestini@aovr.veneto.it

**Keywords:** cancer, physical fitness, quality of life, cardiorespiratory fitness, strength, body fat

## Abstract

Whereas an exercise intervention effectively improves patients’ quality of life, little information is available about the contribution of each physical fitness component. This study aims to explore the association between physical fitness components and the quality-of-life domain in patients with cancer. Between September 2021 and August 2023, 160 patients with mixed cancer types visiting the Oncology Unit were selected on a consecutive basis according to selection criteria. They underwent a comprehensive baseline assessment including the six-minute walking test, the handgrip strength test, the isometric leg press test, the back scratch, sit and reach tests, their waist–hip ratio, and their body mass index. The European Organization for Research and Treatment of Cancer Quality of Life and Core Questionnaire was used to measure the quality of life. The sample size was based on the use of regression models to study associations between clinical characteristics and fitness outcomes. All of the analyses were performed using the SPSS v.25 statistical package. Patients had a mean age of 58 years, 68% were female, 42% were affected by breast cancer, and all were receiving anticancer treatments. Higher functional capacity was associated with better global health status (*p* < 0.0001) and physical (*p* < 0.0001), role (*p* < 0.0001), emotional (*p* = 0.026), and social function (*p* = 0.016) and inversely linked with fatigue (*p* = 0.001). Lower-limb flexibility was significantly associated with all of the domains except for role and social functions. The waist–hip ratio was inversely associated with physical function (*p* < 0.0001) and positively related to fatigue (*p* = 0.037). Exercise programs aiming to improve the quality of life in cancer should be addressed to optimize these fitness components.

## 1. Introduction

Cancer is the leading cause of chronic-disease-related death worldwide, and its burden is set to increase [1]. Over the years, thanks to improvements in medicine, cancer has become an increasingly treatable disease, and more and more innovative anticancer therapies that effectively prolong the survival of patients have been developed [2]. Despite these advances and the increase in life expectancy, patients with cancer may experience an impairment in quality of life (QoL) during their cancer journey. QoL is a complex multidimensional concept that embraces physical, functional, emotional, and social features. The World Health Organization has defined QoL as “an individual’s perception of their position in life in the context of the culture and value systems in which they live and in relation to their goals, expectations, standards, and concerns” [3]. From this point of view, QoL is a multifactorial, subjective, and non-static construct that represents the patients’ appraisal and satisfaction with their current level of functioning compared with what they perceive as the ideal one [4].

A cancer diagnosis is stressful and often accompanied by negative emotions, such as fear, uncertainty, sadness, and anxiety [5]. The disease progression and treatment-related side effects may have negative physical impacts, adversely affecting patients’ daily activities. Additionally, anticancer treatment can last months or even years, leading to a great deal of mental and physical stress involving patients and their social and familiar contexts [6]. All of these situations may compromise the patient’s QoL, with potential negative effects. Indeed, QoL has been found to be a significant prognostic factor in patients with cancer [7]. For instance, an observational study including 6895 patients with mixed cancer types found that the QoL summary score is significantly associated with all-cause mortality even after adjusting for potential covariates. In particular, every 10-point increase in QoL is related to a 23% lower risk of death [8]. Beyond the survival information, QoL also has applications in daily oncology practice [9]. QoL is important in deciding the treatment pathway for patients with advanced incurable diseases. In this context, more value is given to the trade-off between patients’ quality and quantity of life, and the decision-making process is often oriented toward extending the patient’s life without reducing QoL and weighing the risks and benefits of treatment [10].

Therefore, strategies addressed to improve QoL are essential in the cancer context. In this sense, physical exercise appears to have beneficial effects on both quality and quantity of life, helping patients manage the side effects of treatment by reducing fatigue and depressive symptoms on the one hand and increasing survival by reducing mortality rates and recurrence risk on the other [11]. A growing body of evidence has found a positive effect of physical exercise on QoL in oncological patients [12,13]. For instance, a meta-analysis including 16 randomized controlled trials for a total of 877 patients with mixed cancer types found that an exercise program, especially if supervised, is able to significantly increase global health status and physical and social functioning and reduce fatigue compare to the standard of care (mean difference of 5.55 points; 95% CI: 3.19 to 7.90, *p* < 0.001) [14]. Nevertheless, if it is recognized that exercise may improve QoL, less information is currently available about the determinants of QoL related to exercise. Most investigations have been focused on QoL and physical activity, often evaluated through self-reported measures, whereas little is known about the weight of the physical fitness components. Physical fitness is described as “the ability to carry out daily tasks with vigor and alertness, without undue fatigue and with ample energy to enjoy leisure-time pursuits and to meet unforeseen emergencies” [15]. In turn, physical fitness comprises five health-related components, i.e., cardiorespiratory fitness, muscular endurance, muscular strength, body composition, and flexibility, which are particularly important in public health. Identifying the physical fitness factors significantly associated with QoL may help prescribe more tailored physical exercise addressed to improve those components. To our knowledge, few studies have investigated the association between physical fitness and QoL, including patients with breast [16] or colorectal cancer [17,18], or only just one component of health-related fitness. Therefore, the present study aims to explore the association between each physical fitness measure and QoL domains in a sample of patients with mixed cancer types, stages, and treatments.

## 2. Materials and Methods

### 2.1. Study Design and Participants

This cross-sectional analysis reported data collected between 2021 and February 2023. Patient inclusion criteria were (i) age ≥18 years, (ii) a histologically/cytologically confirmed diagnosis of cancer, (iii) >8 weeks from any surgical intervention, (iv) undergoing anticancer treatment, and (v) signed the written informed consent form. Patients were excluded if their clinician did not provide clearance to participate in the study.

The present study adhered to Good Clinical Practice principles, and all of the procedures were conducted in compliance with the Helsinki and Oviedo declarations. The local Ethics Committee for Clinical Trials has reviewed and approved the project (Prot. N. 33320). The present work was carried out following the Strengthening the Reporting Observational studies in Epidemiology (STROBE) guidelines (Supplementary Materials File) [19].

### 2.2. Setting, Procedures and Sampling Method

Potential eligible patients were identified on a consecutive basis and invited to participate in the study by the healthcare providers working at the Oncology Unit of the University of Verona Hospital Trust. If interested, patients signed the informed consent form, and they received an appointment at the gym facility of the Department of Neuroscience, Biomedicine and Movement Science, University of Verona, from the research staff. During the meeting, patients underwent functional assessments with an expert kinesiologist, completed the questionnaires, and provided their medical and sociodemographic data. Data were collected using an Excel spreadsheet (v. 2019).

### 2.3. Health-Related Physical Fitness Evaluation

Health-related physical fitness included the assessment of cardiorespiratory fitness, muscle strength, flexibility, and anthropometric measures [15]. Specialized kinesiologists with certified experience in exercise oncology conducted the assessments using standardized testing protocols and equipment.

#### 2.3.1. Cardiorespiratory Fitness

Cardiorespiratory fitness was estimated through the functional capacity measured using the “Six minutes walking test”, following the standardized protocol of the guidelines of the American Thoracic Society. Patients were instructed to walk at their own pace in a 20 m hallway for six minutes with the aim of covering as much distance as possible. Standard encouragements were given every minute, and the remaining time was called every minute [20]. Contraindications for the test, including a resting heart rate of more than 120, a systolic blood pressure of more than 180 mm Hg, and a diastolic blood pressure of more than 100 mm Hg, were assessed at the start of the evaluation.

#### 2.3.2. Muscle Strength

The handgrip test and the isometric leg press test assessed upper- and lower-limb muscle strength, both measured in kilograms. Regarding the handgrip strength test, we followed a standardized protocol based on the current literature [21,22,23]. To perform the test, the patient sat in a chair with the shoulder adducted, the elbow flexed to 90 degrees, and the wrist aligned with the forearm. Using a hydraulic dynamometer, the patient was asked to perform ten maximum voluntary contractions (five per arm), each separated by thirty seconds of rest. Each contraction was held for 2–4 s until the peak strength no longer increased [23]. The maximal strength reached in the left and right arms was summed. Lower-limb strength was measured using a lead cell horizontally mounted on a leg press machine. The load cell was positioned in series with the sliding axis of the leg press so that the direct line of force could be registered. Patients were seated on the machine with a knee angle of 90 degrees and the seat inclined 30 degrees from the horizontal plane. The patient was instructed to perform five voluntary contractions, kept for 2–4 s, interspersed by 30 s of rest [24]. As suggested by the exercise recommendations of the International Bone Metastases Exercise Working Group [25], patients with bone metastases located in the proximal femur or the spine were excluded from this evaluation. No restriction was adopted for handgrip because bone metastases usually do not affect the forearm bones. Nevertheless, if a metastasis was located in the bones of the forearm or the elbow, the patient was excluded from the handgrip strength test.

#### 2.3.3. Flexibility

For upper-body flexibility, the “back scratch test” was used. This test records how close the hands can be brought together behind the back, attempting to reach or overlap the fingers. A negative score in centimeters was recorded if the middle fingers did not touch each other, whereas it was positive if the middle fingers overlap [26]. Lower-body flexibility was assessed using the “chair sit and reach test”. The patient was sitting on the edge of the chair with a foot flat on the floor, the other leg extended forward, the heel on the floor, and the ankle bent at 90 degrees. The patient was asked to stretch forward as far as possible along the extended leg, trying to reach or overlap the toes with the fingers of the hand [26]. Two trials were performed for both upper- and lower-limb flexibility tests, and the best result in centimeters was recorded.

#### 2.3.4. Anthropometric Measurements

Anthropometric measurements included weight, height, waist, and hip circumferences. Weight and height were measured in light clothes and without shoes, according to the procedures of the World Health Organization [27]. Weight was rounded to the nearest 100 g, whereas height was to the nearest 0.1 cm. Body mass index (BMI) was calculated from the ratio between weight and the square of height (kg/m^2^). Waist circumference was measured at the midpoint between the bottom of the last rib and the top of the hip. The hip circumference was assessed at the maximum circumference above the buttocks. For both measurements, the results were rounded to the nearest 0.1 cm. The waist–hip ratio was calculated by dividing the waist and hip circumference [27].

### 2.4. Quality of Life

Quality of life was assessed using the Italian version of The European Organization for Research and Treatment of Cancer Quality of Life and Core Questionnaire (EORTC QLQ C-30), a questionnaire validated and extensively used in the oncology setting (Supplementary Materials File) [28]. The survey comprised 30 items designed to measure patients’ physical, psychological, and social dimensions of quality of life. Questions are aggregated in a multi-item scale divided into five functional scales (physical, role, cognitive, emotional, and social), three symptom scales (fatigue, pain, nausea, and vomiting), a global health and quality-of-life scale, and the perceived financial impact of the disease. The remaining six single items investigate additional symptoms commonly experienced by patients with cancer (dyspnea, appetite loss, sleep disturbance, constipation, and diarrhea) [28]. Each question is rated from 1 (not at all) to 4 (very much), and all scale items are then transformed into a score ranging from 0 to 100. Higher scores on the symptom scale indicate more symptom burden, whereas higher scores on the functional scales represent higher functioning levels.

### 2.5. Covariates 

A self-administered questionnaire was used to collect sociodemographic data and physical activity levels (Supplementary Materials File). Sociodemographic data included age (years), gender (male/female), education level (elementary up to 10–11 years/secondary up to 14 years/secondary up to 18–19 years/college or university/postgraduate), marital status (single/married/divorced/widowed/other), employment (full-time employment/part-time employment/retired/homemaker/other), and the adequacy of financial resources (more than adequate/adequate/barely adequate/inadequate). The current physical activity level was assessed using Godin’s Leisure Time Exercise Questionnaire [29]. This questionnaire asks about the previous week’s leisure time frequency and duration of vigorous-, moderate-, and mild-intensity exercise. Medical variables were recorded using interview questions and included the cancer site, cancer stage, date of diagnosis, and type and duration of treatment. Patients were divided into two subgroups based on the time since diagnosis using a median of 30 months as the cutoff [30].

### 2.6. Statistical Analysis and Sample Size

The number of patients evaluated was determined to study associations between fitness measurements, QoL, and patients’ characteristics. Considering 8 covariates to adjust estimations, a sample size of 160 patients has a power of 90% at a significance level of 0.006 (global *p* value of 5% divided by 8 independent variables to be tested) to assess an effect size f^2^ = R^2^/(1 − R^2^) equal to 0.10, an effect size considered between small and medium. 

Descriptive statistics are presented as the mean, standard deviation, medians, and interquartile range for continuous variables, whereas frequencies and percentages are used for categorical variables. Linear regression was used to investigate the physical fitness parameters associated with QoL domains. Firstly, the unadjusted model was created for each physical fitness test. A second model was adjusted for age, sex, moderate/vigorous exercise level, education, marital status, employment, financial resources, cancer type, and stage. Beta coefficients and 95% confidence interval (CI) are used to present the results and the AIC (Akaike Information Criterion) is reported. The AIC is a measure that balances between model fit and complexity in terms of the number of variables considered. Statistical tests were two-sided, and *p*-values < 0.05 were considered significant. The SPSS statistical package (version 25, IBM, Armonk, NY, USA) was used.

## 3. Results

### 3.1. Characteristics of the Study Participants

**Figure 1:**
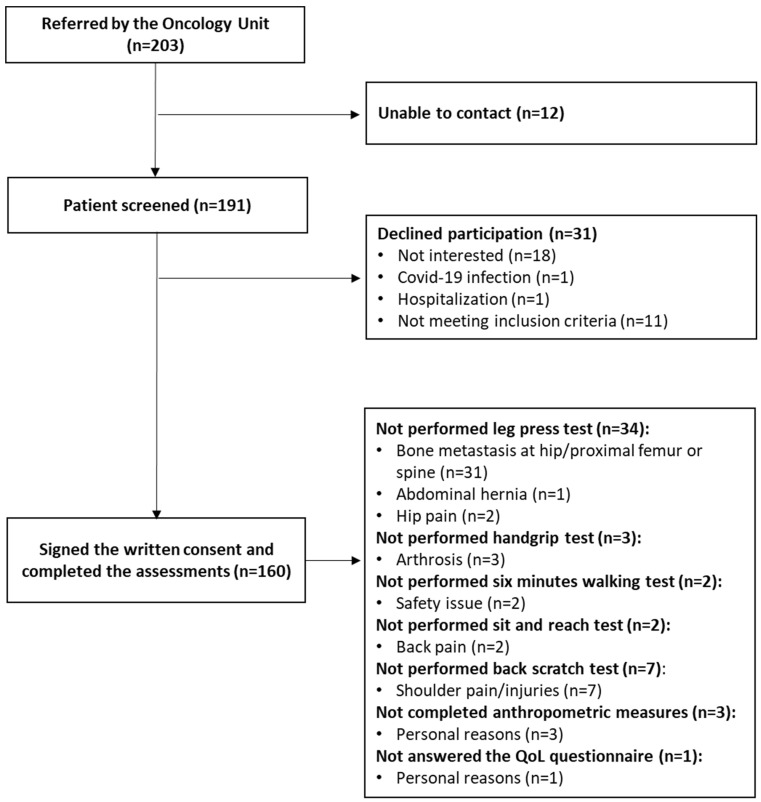
Study flow diagram.

Among the 203 referred patients (Figure 1), 160 patients participated in the study. The clinical and sociodemographic parameters of the patients are shown in Table 1. Overall, 68.7% of the patients were female, and the mean age was 58.83 ± 10.56 years. Most patients were married (76.3%) and had a high school level of education (39.4%). Regarding medical variables, 42.1% of the participants had breast cancer, and 31.0% had gastrointestinal cancer. All of the patients were still on active treatments during the assessments, 62.5% underwent surgery, and 71.9% received chemotherapy.

Patients’ physical fitness, current level of exercise, and QoL variables are collected in Table 2. Thirty-four patients did not perform the leg press test due to the presence of bone metastases (n = 31), abdominal hernia (n = 1), and hip pain (n = 2), whereas other patients did not perform evaluations of cardiorespiratory fitness (n = 2), upper-limb strength (n = 3), and flexibility (n = 9) due to safety issues. One patient did not complete the QoL questionnaire, and three did not complete anthropometric measures for personal reasons.

Briefly, patients reported a mean BMI of 26.00 kg/m^2^ and a mean waist–hip ratio equal to 0.88 cm. Regarding physical tests, mean values of 506.7 m in the 6MWT, 57.3 kg for upper limbs, and 84.6 kg for lower limbs were registered. In the back scratch test, a mean value of −3.6 cm for the right arm and −8.5 for the left arm was observed, whereas in the chair sit and the reach test, the mean value found was −2.8 cm. Regarding QoL, the global health status reached a mean value of 62.8 points and physical and cognitive functioning was the scale with the highest values, 82.0 and 84.7 points, respectively, while among the symptoms, fatigue (35.0 points), insomnia, and dyspnea (25.4 points) were the most reported.

### 3.2. Relationship between Physical Fitness and Quality of Life

Table 3 reports the physical fitness parameters associated with QoL domains. In the unadjusted model, a higher 6MWT was significantly associated with better global health status (β = 0.065; 95% CI: 0.037 to 0.092; *p* < 0.0001) and physical (β = 0.080; 95% CI: 0.060 to 0.101; *p* < 0.0001), role (β = 0.078; 95% CI: 0.043 to 0.114; *p* < 0.0001), and social function (β = 0.044; 95% CI: 0.010 to 0.078; *p* = 0.012) and inversely related with fatigue (β = −0.043; 95% CI: −0.076 to −0.010; *p* = 0.010). A significant association was also detected for upper-limb strength with physical function (β = 0.244; 95% CI: 0.103 to 0.385; *p* < 0.0001) and fatigue (β = −0.302; 95% CI: −0.499 to −0.106; *p* = 0.003) and for lower-limb strength and physical (β = 0.058; 95% CI: 0.006 to 0.111; *p* = 0.030) and social function (β = −0.095; 95% CI: −0.172 to −0.017; *p* = 0.016). The lower-limb flexibility was associated with emotional function (β = 0.317; 95% CI: 0.042 to 0.593; *p* = 0.024) and the waist–hip ratio with physical function (β = −33.778; 95% CI: −57.386 to −10.169; *p* = 0.005), whereas the back scratch test and BMI did not show a significant association. After adjusting for potential covariates, 6MWT remained positively associated with the domains found in the unadjusted model (global health status: β = 0.079; 95% CI: 0.046 to 0.113; *p* < 0.0001; physical function: β = 0.080; 95% CI: 0.055 to 0.104; *p* < 0.0001; role function: β = 0.099; 95% CI: 0.055 to 0.143; *p* < 0.0001; social function: β = 0.051; 95% CI: 0.010 to 0.093; *p* = 0.016; fatigue: β = −0.063; 95% CI: −0.100 to −0.025; *p* = 0.001), and, additionally, a significative association with emotional function (β = 0.044; 95% CI: 0.005 to 0.083; *p* = 0.026) emerged. The relationship between upper-body strength and physical function (β = 0.274; 95% CI: 0.070 to 0.477; *p* = 0.008) persisted. Lower-limb strength was inversely associated with social function (β = −0.101; 95% CI: −0.192 to −0.011; *p* = 0.028). Lower-limb flexibility remained associated with emotional function (β = 0.363; 95% CI: 0.071 to 0.656; *p* = 0.015), global health status (β = 0.277; 95% CI: 0.009 to 0.545; *p* = 0.043) and physical (β = 0.231; 95% CI: 0.025 to 0.436; *p* = 0.028) and cognitive function (β = 0.350; 95% CI: 0.109 to 0.591; *p* = 0.004) and negatively related to fatigue (β = −0.406; 95% CI: −0.695 to −0.116; *p* = 0.006). The waist–hip ratio was still inversely associated with physical function (β = −42.537; 95% CI: −67.759 to −17.316; *p* < 0.0001), and a positive association with fatigue (β = 38.684; 95% CI: 2.349 to 75.020; *p* = 0.037) was detected. In general, models assessing the associations between physical fitness parameters and physical functioning evaluated using the questionnaire show the best (i.e., the minimum) AICs.

## 4. Discussion

This cross-sectional study explored the associations between QoL domains and physical fitness parameters of patients with cancer receiving anticancer treatments. Greater cardiorespiratory fitness, estimated using the 6MWT, was positively associated with better global health status and physical, role, emotional, and social functions and inversely linked to fatigue after adjusting the model for potential confounding factors.

A prior investigation conducted on patients affected by colorectal cancer, while confirming the linking between cardiorespiratory fitness, fatigue, and functional scales, did not find a significant association with global health status [17]. Although the precise reasons for this discrepancy are currently unknown, our results add another piece to reinforce the importance of cardiorespiratory fitness in the oncological context. Indeed, besides being a determinant of QoL, cardiorespiratory fitness is a strong independent predictor of overall, cancer-specific, and cardiovascular mortality in patients with cancer after adjusting for potential clinical covariates [31]. Therefore, our findings and those available from the literature suggest that increasing cardiorespiratory fitness may directly impact patients’ quantity and quality of life. During assessments in our investigation, all of the patients were currently receiving anticancer treatments, mainly chemotherapy and hormonal therapy, and more than half had undergone surgery (62.5%). These patients inevitably experience a loss in cardiorespiratory fitness ranging from 5 to 26%, potentially harming their QoL [32]. A recent reference standard from a healthy population sample is 581.4  ±  66.5 m for females and 608.7  ±  80.1 m for males, thus confirming that functional capacity can be impaired in patients with cancer undergoing treatments [33]. Physical exercise is the optimal strategy for increasing cardiorespiratory fitness, and its impact on this endpoint has been evaluated in different meta-analyses. Scott and colleagues included 48 randomized controlled trials predominantly involving patients in the early stages of disease, and the results show that exercise was able to increase the peak oxygen consumption by +2.80 mLO_2_ × kg^−1^ × min^−1^ whereas the controls remained stable (+0.02 mLO_2_ × kg^−1^ × min^−1^) [32]. In the advanced-stage setting, data are more limited; nevertheless, a meta-analysis including ten randomized trials found a weighted mean difference between exercise and control groups of +20.86 m in the 6MWT in favor of exercise [34].

As reported in previous studies [17,18,35], we observed an inverse association between cardiorespiratory fitness and fatigue, and, additionally, the waist–hip ratio and lower-limb flexibility were associated with fatigue. This is not surprising as the role of exercise in improving cancer-related fatigue is well-established with respect to different other interventions. For instance, a meta-analysis including 113 studies and 11,525 patients compared the effect of pharmacological, exercise, and psychological interventions to treat fatigue. Whereas pharmacological interventions produced only a small effect, exercise plus psychological approaches generated a moderate effect, and exercise alone demonstrated the largest overall improvement in ameliorating cancer-related fatigue [36]. In this sense, the contribution of exercise is so crucial that the European Society of Medical Oncology (ESMO) reported it as the first line of treatment for cancer-related fatigue [37]. Thus, although it is known that an exercise intervention improves fatigue, our work may contribute to explaining the pathway through which it acts, i.e., through an increase in cardiorespiratory fitness and flexibility and the modulation of abdominal fat. High cardiorespiratory fitness reflects a better ability of the cardiorespiratory, vascular, and skeletal systems to transport and use oxygen during exercise or more general activities. By increasing the cardiorespiratory reserve, the body becomes more efficient and cheap in performing the activities of daily living, probably also resulting in a minor perception of the exertion. A similar hypothesis could be made for abdominal fat assessed through the waist–hip ratio: greater abdominal fat could make it more difficult to perform daily living activities. According to the World Health Organization’s reference values, a waist–hip ratio >0.85 in women and >0.9 in men corresponds to an overweight BMI (i.e., 25–29.9 kg/m^2^) and is considered high [38]. Another pathway through which cardiorespiratory fitness and abdominal fat may act is the inflammatory status. Indeed, cancer-related fatigue is characterized by a high inflammatory status [39]. Different studies show that low cardiorespiratory fitness and body fat are correlated with higher levels of inflammatory biomarkers, thus reinforcing the speculation that just these features may drive the improvement in fatigue [40]. 

Moreover, anthropometric measures revealed an association between abdominal fat and physical function, while BMI did not show any relation to QoL. Prior investigations have found similar results [35], whereas others found mixed findings [41,42,43]. Generally, obesity, assessed through BMI, appears to have the greatest negative impact on QoL in cancer compared to overweight and normal weight [41,42,43]. Nevertheless, our study did not detect any link to this, probably due to the assessment methods and the mixed included population. Additionally, whereas the World Health Organization classified obesity as a BMI ≥30 kg/cm^2^ [44], our sample had a mean BMI of 26 kg/m^2^, which is at the lower limit of overweight. Thus, it is possible that the link between BMI and QoL would be more evident in a sample of obese patients. Body composition is a *hot topic* in oncology. The obesity paradox, i.e., the speculation that overweight confers overall survival advantages, is confirmed in some cancer types, such as pancreatic, lung, and colorectal cancer [45], but not in others, including breast and endometrial [46]. Although the association between obesity and survival benefits may seem unintuitive given the recognized negative impact of body fatness on health, this may be explained by the fact that BMI may not be the optimal tool to utilize in this setting. BMI has low sensitivity in identifying obesity, and it cannot differentiate between fat and muscle mass. In addition, many cancers, as a consequence of the disease itself and/or the treatments, result in significant weight loss, predominantly in muscle mass, with an associated deterioration of QoL [47]. The different features of body composition, i.e., fat and muscle mass, also play different roles in QoL. Indeed, a meta-analysis including 14 studies and 2776 patients found that low skeletal muscle mass is inversely associated with global health status and physical functioning [48], thus suggesting that deepening the evaluation of body composition as a determinant of QoL is necessary.

We assessed the impact of upper- and lower-limb flexibility as determinants of QoL. Whereas the upper-body test was not related to any QoL domain, significant associations between lower-limb flexibility and global health status, physical, emotional, and cognitive function, and fatigue emerged. Flexibility is an essential feature in maintaining activities of daily living with an adequate range of motion, and trials focused on stretching, Pilates, or yoga (mind–body interventions) are largely used for improving the QoL of patients with cancer [49,50,51,52]. Stretching is considered a very low-intensity exercise and is often recommended to improve body flexibility and overall mood.

Contrary to our expectations, muscle strength was only slightly correlated with QoL. The strength of lower limbs results in being inversely associated with social function, while the handgrip strength test exhibited a positive association with physical function. Prior research has reported different results. On the one hand, handgrip strength was related to fatigue and QoL in patients with breast cancer [53] and pain/discomfort in Korean cancer survivors [54]. On the other hand, similarly to our results, two investigations have reported a significant association between strength and physical function in patients with colorectal [17] and mixed cancer types [55]. These findings suggest a link between strength and QoL, even if the inconsistency in QoL domains exists. Clearly, heterogeneity in the study sample across the investigations may explain these differences. Indeed, it may be possible that muscle strength could differently affect QoL domains based on the cancer type and tr..eatment status. Future research should verify these hypotheses. Moreover, criticisms about strength assessments may arise. Indeed, muscle strength may be evaluated in several ways. The maximum repetition test is submaximal, and it is a relatively safe procedure that can be carried out for most muscle groups, but it has the risk of praxis adaptation [56]. The handgrip strength test has the advantage of requiring portable and relatively inexpensive equipment, such as a dynamometer. However, because only small amount of muscle mass is activated, the generalizability of such an evaluation as a measurement of overall strength is limited [56]. Finally, the isokinetic/isometric and maximal voluntary contraction tests are maximal tests, and they are considered the gold standard for evaluating strength [56]. Nevertheless, in addition to the need for specific and expensive equipment, concerns about safety in some cancer populations may limit their applicability. In patients with bone metastases, assessment directly involving the bone lesion could be risky, and, to date, even if specific recommendations for standardized approaches for testing this population are not available, the benefit–risk ratio should always be weighted [25]. For instance, as in our study, patients with bone metastasis in the proximal femur could be excluded from leg press testing because the injured part is directly involved in the movement. Despite the beneficial role of strength in patients with cancer to perform activities of daily living, maintain autonomy, and increase muscle mass [57] and as a predictor of survival [58], its contribution to QoL necessitates further investigations that address the issues just mentioned.

This study’s limitations should be noted. Firstly, the cross-sectional design does not make it possible to identify causal inferences. Secondly, the small sample size, together with the heterogeneous cancer population in terms of cancer type, disease stage, and treatments, may limit the generalizability of our findings. Nevertheless, the strength of this study is the use of standardized, validated, and widely utilized fitness tests, which allowed us to have a realistic estimation of the patient’s physical fitness. In addition, to the best of our knowledge, this is the first study assessing the association between each physical fitness component and QoL domains in a group of patients with mixed cancers. Although our results are not generalizable, they provide useful data to develop future exercise interventions to improve QoL in this population.

## 5. Conclusions 

In conclusion, physical fitness is associated with different domains of QoL in patients with cancer. Among the different features of fitness, cardiorespiratory fitness and lower-limb flexibility were the most associated with a better QoL and a reduction in cancer-related fatigue. Whereas these results support the importance of physical exercise in the cancer context, they may also provide guidance for exercise professionals in prescribing programs aimed at improving quality of life, emphasizing the importance of patient-centered approaches. Because QoL is both a crucial outcome in clinical practice and a prognostic factor, this study could also be valuable for healthcare providers who need to understand the factors that influence QoL and the strategies to improve it. Future research could focus on exploring if the weight of physical fitness components contributing to QoL may differ based on cancer types or treatment, as well as investigating the impact of other physical variables (e.g., muscle mass, bone density).

## Figures and Tables

**Table 1:** Baseline characteristics of the study participants.

Variable	No.	%
Sex		
Male	50	31.3
Female	110	68.7
Education		
Elementary (up to 10–11 years)	2	1.3
Secondary (up to 14 years)	44	27.5
Secondary (up to 18–19 years)	63	39.4
College/university	39	24.4
Post-graduated	11	6.9
Missing	1	0.5
Marital status		
Single	17	10.6
Married	122	76.3
Divorced	15	9.4
Widowed	3	1.9
Other	1	0.6
Missing	2	1.3
Employment		
Part-time employment	22	13.8
Full-time employment	50	31.3
Homemaker	6	3.8
Retired	55	34.2
Other	27	16.2
Missing	1	0.6
Financial resources		
Inadequate	4	2.5
Barely adequate	30	18.8
Adequate	90	56.3
More than adequate	35	21.9
Missing	1	0.6
Cancer site		
Breast	72	42.1
Thoracic	23	13.5
Gynecologic/genitourinary/other	23	13.5
Gastrointestinal	53	31.0
Cancer stage		
I	37	23.1
II	24	15.0
III	35	21.9
IV	64	40.0
Time from diagnosis		
≥30 months	35	21.9
<30 months	125	78.1
Type of treatment		
Chemotherapy	115	71.9
Radiotherapy	10	6.3
Surgery	100	62.5
Immunotherapy	5	3.1
Target therapy	20	12.5
Hormone therapy	68	42.5

**Table 2:** Physical fitness and quality of life variables of patients.

Variable	No.	Mean (SD)	Median (IQR)
Cardiorespiratory fitness (6MWT—meter)	158	506.7 (100.1)	517.4 (452.0–574.0)
Upper strength (handgrip strength test—kg)	157	57.3 (16.5)	54.0 (45.0–66.0)
Lower strength (Leg press—kg)	126	84.6 (49.7)	74.1 (55.0–103.0)
Anthropometric measures			
Body mass index (kg/m^2^)	157	26.00 (5.21)	25.26 (22.31–28.41)
Waist–hip ratio (cm)	159	0.88 (0.10)	0.89 (0.80–0.95)
Flexibility			
Sit and reach (cm)	158	−2.8 (11.5)	−1.0 (−12.0–4.0)
Back scratch right arm (cm)	156	−3.6 (10.2)	0 (−10.0–4.0)
Back scratch left arm (cm)	157	−8.5 (10.6)	−7.0 (−16.0–0)
Physical activity level (min/week)			
Vigorous	160	26.8 (89.2)	0 (0–0)
Moderate	160	123.6 (233.1)	0 (0–160.0)
Light	160	229.9 (472.9)	37.5 (0–240.0)
Quality of life (EORTC QLQ-C30)			
Physical functioning	159	82.0 (15.4)	86.7 (73.3–93.3)
Role functioning	159	75.8 (24.2)	83.3 (66.7–100)
Emotional functioning	159	73.6 (20.6)	75.0 (66.7–91.7)
Cognitive functioning	159	84.7 (17.3)	83.3 (83.3–100)
Social functioning	159	73.2 (22.3)	66.7 (66.7–100)
Global health status	159	62.8 (19.0)	66.7 (50.0–75.0)
Fatigue	159	35.0 (21.3)	33.3 (22.2–44.4)
Nausea/vomiting	159	8.2 (16.3)	0 (0–16.67)
Pain	159	21.6 (21.3)	16.7 (0–33.3)
Dyspnea	159	25.4 (23.8)	33.3 (0–33.3)
Insomnia	159	25.4 (23.8)	33.3 (0–33.3)
Appetite loss	159	14.7 (25.6)	0 (0–33.3)
Constipation	159	14.9 (23.3)	0 (0–33.3)
Diarrhea	159	8.8 (18.5)	0 (0–0)
Financial problems	159	11.1 (21.8)	0 (0–16.7)

Abbreviations: EORTC QLQ-C30, European Organization for the Research and Treatment of Cancer Quality of Life Questionnaire-Core 30; IQR, interquartile range; SD, standard deviation; 6MWT, six-minute walking test.

**Table 3:** Regression modelling of associations of physical fitness parameters with quality-of-life domains.

	6MWT			Sit and Reach			Back Scratch Right Arm			Back Scratch Left Arm		
	β (95% CI)	*p*-Value	AIC	β (95% CI)	*p*-Value	AIC	β (95% CI)	*p*-Value	AIC	β (95% CI)	*p*-Value	AIC
Unadjusted Model												
Global health status	0.065 (0.037; 0.092)	<0.0001 *	1356.6	0.224 (−0.030; 0.478)	0.085	1371.0	0.077 (−0.213; 0.366)	0.604	1353.6	0.103 (−0.176; 0.382)	0.468	1361.0
Physical function	0.080 (0.060; 0.101)	<0.0001 *	1260.3	0.187 (−0.018; 0.392)	0.074	1303.6	0.158 (−0.078; 0.394)	0.190	1290.5	0.192 (−0.035; 0.419)	0.097	1296.9
Role function	0.078 (0.043; 0.114)	<0.0001 *	1435.6	0.233 (−0.094; 0.560)	0.163	1450.1	0.162 (−0.201; 0.526)	0.382	1424.7	0.163 (−0.189; 0.516)	0.363	1433.7
Emotional function	0.020 (−0.012; 0.052)	0.218	1400.4	0.317 (0.042; 0.593)	0.024 *	1395.9	0.053 (−0.267; 0.372)	0.747	1384.2	−0.027 (−0.335; 0.282)	0.866	1392.4
Cognitive function	0.012 (−0.015; 0.039)	0.387	1345.1	0.189 (−0.044; 0.423)	0.112	1344.1	0.034 (−0.235; 0.302)	0.805	1330.7	0.144 (−0.115; 0.403)	0.275	1337.9
Social function	0.044 (0.010; 0.078)	0.012 *	1420.4	0.258 (−0.041; 0.558)	0.091	1422.4	0.161 (−0.181; 0.504)	0.355	1405.8	0.090 (−0.242; 0.423)	0.595	1415.9
Fatigue	−0.043 (−0.076; −0.010)	0.010 *	1406.0	−0.211 (−0.498; 0.077)	0.151	1409.6	0.225 (−0.103; 0.554)	0.178	1392.7	0.133 (−0.185; 0.451)	0.414	1401.9
Adjusted Model												
Global health status	0.079 (0.046; 0.113)	<0.0001 *	1356.4	0.277 (0.009; 0.545)	0.043 *	1374.8	0.128 (−0.183; 0.439)	0.419	1356.4	0.161 (−0.147; 0.469)	0.306	1363.7
Physical function	0.080 (0.055; 0.104)	<0.0001 *	1263.2	0.231 (0.025; 0.436)	0.028 *	1292.0	0.170 (−0.073; 0.412)	0.172	1279.9	0.184 (−0.057; 0.424)	0.134	1286.6
Role function	0.099 (0.055; 0.143)	<0.0001 *	1447.1	0.300 (−0.053; 0.654)	0.096	1461.7	0.152 (−0.246; 0.551)	0.454	1433.1	0.166 (−0.231; 0.563)	0.413	1443.0
Emotional function	0.044 (0.005; 0.083)	0.026 *	1403.8	0.363 (0.071; 0.656)	0.015 *	1402.7	0.163 (−0.185; 0.510)	0.358	1390.6	0.121 (−0.224; 0.466)	0.491	1398.8
Cognitive function	0.018 (−0.014; 0.050)	0.278	1348.4	0.350 (0.109; 0.591)	0.004 *	1341.8	0.143 (−0.147; 0.433)	0.333	1344.3	0.275 (−0.010; 0.560)	0.058	1339.5
Social function	0.051 (0.010; 0.093)	0.016 *	1428.2	0.283 (−0.035; 0.601)	0.081	1428.4	0.169 (−0.202; 0.540)	0.373	1411.2	0.079 (−0.292; 0.449)	0.678	1421.6
Fatigue	−0.063 (−0.100; −0.025)	0.001 *	1396.8	−0.406 (−0.695; −0.116)	0.006 *	1399.2	0.076 (−0.267; 0.418)	0.664	1386.1	−0.051 (−0.390; 0.289)	0.770	1393.9

Adjusted for age, sex, cancer type stage, moderate/vigorous exercise level, education, marital status, employment, and financial resources. CI: confidence interval; 6MWT, six minutes walking test; AIC: Akaike Information Criterion. * Statistically significant.

## Data Availability

Data are available from the corresponding author upon reasonable request.

## Supplementary Materials

The following supporting information can be downloaded at: https://www.mdpi.com/article/10.3390/healthcare12161643/s1. Supplementary Materials File (S1. STROBE Statement—checklist of items that should be included in reports of cross-sectional studies; S2. Questionnaire for sociodemographic data; S3. Godin’s Leisure Time Exercise Questionnaire; S4. European Organization for Research and Treatment of Cancer Quality of Life and Core Questionnaire (EORTC QLQ C-30)).
