# Supplementary material for: Exploring the Association between Health-Related Physical Fitness and Quality of Life in Patients with Cancer: A Cross-Sectional Study
Source: Healthcare (Basel). 2024 Aug 17;12(16):1643. doi: 10.3390/healthcare12161643 (PMC11353676; doi:10.3390/healthcare12161643)
Supplement: Supplementary file 1 [file healthcare-12-01643-s001.zip › healthcare-3144209-supplementary.pdf]

Supplementary material

S1. STROBE Statement—Checklist of items that should be included in reports of ***cross-sectional studies***

S2. Questionnaire for sociodemographic data

S3. Godin's Leisure Time Exercise Questionnaire

S4. European Organization for Research and Treatment of Cancer Quality of Life and Core Questionnaire (EORTC QLQ C-30)

S1. STROBE Statement—Checklist of items that should be included in reports of ***cross-sectional studies***

	Item No	Recommendation	Page No
Title and abstract	1	(*a*) Indicate the study's design with a commonly used term in the title or the abstract	1
		(*b*) Provide in the abstract an informative and balanced summary of what was done and what was found	1
Introduction			
Background/rationale	2	Explain the scientific background and rationale for the investigation being reported	2-3
Objectives	3	State-specific objectives, including any prespecified hypotheses	2-3
Methods			
Study design	4	Present key elements of study design early in the paper	3
Setting	5	Describe the setting, locations, and relevant dates, including periods of recruitment, exposure, follow-up, and data collection	3
Participants	6	(*a*) Give the eligibility criteria, and the sources and methods of selection of participants	3
Variables	7	Clearly define all outcomes, exposures, predictors, potential confounders, and effect modifiers. Give diagnostic criteria, if applicable	3-5
Data sources/ measurement	8	For each variable of interest, give sources of data and details of methods of assessment (measurement). Describe comparability of assessment methods if there is more than one group	3-4
Bias	9	Describe any efforts to address potential sources of bias	5-14
Study size	10	Explain how the study size was arrived at	5
Quantitative variables	11	Explain how quantitative variables were handled in the analyses. If applicable, describe which groupings were chosen and why	5
Statistical methods	12	(*a*) Describe all statistical methods, including those used to control for confounding	5
		(*b*) Describe any methods used to examine subgroups and interactions	5
		(*c*) Explain how missing data were addressed	5
		(*d*) If applicable, describe analytical methods taking account of sampling strategy	5
		(*e*) Describe any sensitivity analyses	5
Results			
Participants	13	(a) Report numbers of individuals at each stage of study—eg numbers potentially eligible, examined for eligibility, confirmed eligible, included in the study, completing follow-up, and analysed	5
		(b) Give reasons for non-participation at each stage	5
		(c) Consider use of a flow diagram	6
Descriptive data	14	(a) Give characteristics of study participants (eg demographic, clinical, social) and information on exposures and potential confounders	6-8
		(b) Indicate number of participants with missing data for each variable of interest	8
Outcome data	15	Report numbers of outcome events or summary measures	8-9
Main results	16	(*a*) Give unadjusted estimates and, if applicable, confounder-adjusted estimates and their precision (eg, 95% confidence interval). Make clear which confounders were adjusted for and why they were included	8-10

		(*b*) Report category boundaries when continuous variables were categorized	8-10
		(*c*) If relevant, consider translating estimates of relative risk into absolute risk for a meaningful time period	8-10
Other analyses	17	Report other analyses done—eg analyses of subgroups and interactions, and sensitivity analyses	8-10
**Discussion**			
Key results	18	Summarise key results with reference to study objectives	12
Limitations	19	Discuss limitations of the study, taking into account sources of potential bias or imprecision. Discuss both direction and magnitude of any potential bias	14
Interpretation	20	Give a cautious overall interpretation of results considering objectives, limitations, multiplicity of analyses, results from similar studies, and other relevant evidence	12-14
Generalisability	21	Discuss the generalisability (external validity) of the study results	12-14
**Other information**			
Funding	22	Give the source of funding and the role of the funders for the present study and, if applicable, for the original study on which the present article is based	15

S2. Questionnaire for sociodemographic data

1. Date of birth: ______________________
2. Sex: □ male □ female
3. Education level: □ elementary (up to age 10-11 years) □ secondary (up to age 14 years) □ secondary (up to age 18-19 years) □ college/university □ postgraduate 7. Marital status: □ single □ married □ divorced □ widow/widower
4. Employment: □ retired □ homemaker □ part-time employed □ full-time employed □ other
5. Does your monthly income cover your monthly expenditure? □ inadequate □ barely adequate □ adequate □ more than adequate

S3. Godin's Leisure Time Exercise Questionnaire

During your leisure time, over the **last week** (last 7 days), on average how many times do you engage in the following types of physical exercise? Please indicate the weekly frequency (number of days per week) and the time dedicated for ONE of these days.		
Type	Frequency (days a week)	Duration (average duration per session)
LOW-INTENSITY PHYSICAL ACTIVITY (minimal effort, an activity that allows you to speak comfortably while performing it) E.g., yoga, walking, etc.	n.________________	Min._________
MODERATE-INTENSITY PHYSICAL ACTIVITY (not exhausting, an activity that allows you to talk with some difficulty while performing it) E.g., brisk walking, cycling, dancing, etc.	n.________________	Min._________
HIGH-INTENSITY PHYSICAL ACTIVITY (corresponds to a high heart rate, an activity that does NOT allow you to talk while performing it) E.g., running, jogging, etc.	n.________________	Min._________

S4. European Organization for Research and Treatment of Cancer Quality of Life and Core Questionnaire (EORTC QLQ C-30)

During the past week:		Not at all	A little	Quite a bit	Very much
1	Do you have any trouble doing strenuous activities, like carrying a heavy shopping bag or a suitcase?	1	2	3	4
2	Do you have any trouble taking a long walk?	1	2	3	4
3	Do you have any trouble taking a short walk?	1	2	3	4
4	Do you need to stay in bed or a chair during the day?	1	2	3	4
5	Do you need help with eating, dressing, washing yourself or using the toilet?	1	2	3	4
6	Were you limited in doing either your work or other daily activities?	1	2	3	4
7	Were you limited in pursuing your hobbies or other leisure time activities?	1	2	3	4
8	Were you short of breath?	1	2	3	4
9	Have you had pain?	1	2	3	4
10	Did you need to rest?	1	2	3	4
11	Have you had trouble sleeping?	1	2	3	4
12	Have you felt weak?	1	2	3	4
13	Have you lacked appetite?	1	2	3	4
14	Have you felt nauseated?	1	2	3	4
15	Have you vomited?	1	2	3	4
16	Have you been constipated?	1	2	3	4
17	Have you had diarrhea?	1	2	3	4
18	Were you tired?	1	2	3	4
19	Did pain interfere with your daily activities?	1	2	3	4
20	Have you had difficulty in concentrating on things, like reading a newspaper or watching television?	1	2	3	4
21	Did you feel tense?	1	2	3	4
22	Did you worry?	1	2	3	4
23	Did you feel irritable?	1	2	3	4
24	Did you feel depressed?	1	2	3	4
25	Have you had difficulty remembering things?	1	2	3	4

<table>
<tr><td>26</td><td colspan="2">Has your physical condition or medical treatment interfered with your family life?</td><td>1</td><td>2</td><td>3</td><td>4</td></tr>
<tr><td>27</td><td colspan="2">Has your physical condition or medical treatment interfered with your social activities?</td><td>1</td><td>2</td><td>3</td><td>4</td></tr>
<tr><td>28</td><td colspan="2">Has your physical condition or medical treatment caused you financial difficulties?</td><td>1</td><td>2</td><td>3</td><td>4</td></tr>
<tr><td colspan="7">For the following questions please circle the number between 1 and 7 that best applies to you</td></tr>
<tr><td>29</td><td colspan="6">How would you rate your overall health during the past week?</td></tr>
<tr><td>1</td><td>2</td><td>3</td><td>4</td><td>5</td><td>6</td><td>7</td></tr>
<tr><td>Very poor</td><td></td><td></td><td></td><td></td><td></td><td>Excellent</td></tr>
<tr><td>30</td><td colspan="6">How would you rate your overall quality of life during the past week?</td></tr>
<tr><td>1</td><td>2</td><td>3</td><td>4</td><td>5</td><td>6</td><td>7</td></tr>
<tr><td>Very poor</td><td></td><td></td><td></td><td></td><td></td><td>Excellent</td></tr>
</table>
